# An EV-Guided Multi-Compartment Proof-of-Concept Framework for Biomarker Prioritization in Cholangiocarcinoma

**DOI:** 10.3390/medsci14010122

**Published:** 2026-03-05

**Authors:** Kanawut Kotawong, Sittiruk Roytrakul, Narumon Phaonakrop, Kesara Na-Bangchang, Wanna Chaijaroenkul

**Affiliations:** 1 Center of Excellence in Pharmacology and Molecular Biology of Malaria and Cholangiocarcinoma, Chulabhorn International College of Medicine, Thammasat University (Rangsit Campus), 99 Moo 18 Phaholyothin Road, Klong Luang District, Pathumthani 12120, Thailand; kanawut.mt@gmail.com (K.K.); kesaratmu@yahoo.com (K.N.-B.); 2 Functional Proteomics Technology Laboratory, National Center for Genetic Engineering and Biotechnology, National Science and Technology Development Agency, Pathumthani 12120, Thailand; sittiruk2000@gmail.com (S.R.); narumon.pha@biotec.or.th (N.P.)

**Keywords:** cholangiocarcinoma, extracellular vesicles, EV proteomics, biomarker prioritization, multi-compartment analysis

## Abstract

Background: Cholangiocarcinoma (CCA) is a highly heterogeneous malignancy in which numerous biomarker candidates have been reported, yet few progress to clinical use. Beyond biological complexity, this low translational yield reflects the lack of systematic criteria for prioritizing biomarkers during the discovery stage. In particular, tumor-derived signals identified in tissue often fail to persist in clinically accessible biofluids, as cross-compartment signal behavior is rarely evaluated explicitly. Methods: We developed an extracellular vesicle (EV)-guided, multi-compartment proof-of-concept framework to assess biomarker robustness and translatability early in discovery. EV proteomes from three biologically distinct CCA cell lines and a normal cholangiocyte were analyzed using multivariate and machine-learning-assisted approaches to identify conserved EV-associated features. These were integrated with public transcriptomic, epigenetic, copy-number, promoter usage, and miRNA regulatory data. Tissue relevance was assessed using TCGA/GTEx RNA-seq datasets, and exploratory signal behavior was examined in pooled serum- and urine-derived EVs from CCA patients and controls. Results: EV proteomics revealed marked molecular heterogeneity across CCA models but identified a small subset of conserved EV-associated proteins. SERPINF2 was used as a representative example, showing consistently reduced EV-associated abundance across all CCA models with coordinated regulation across multiple molecular layers. SERPINF2 expression was independent of patient sex and tumor stage and clearly distinguished tumor from normal bile duct tissue. Exploratory biofluid analyses demonstrated compartment-dependent signal behavior, with SERPINF2 depletion detectable in urine-derived EVs but not in serum-derived EVs. Conclusions: Rather than validating a single biomarker, this study presents an EV-guided, multi-compartment framework for prioritizing biomarker candidates at the discovery stage. By explicitly accounting for tumor heterogeneity and compartment-specific signal preservation, this proof-of-concept approach provides a practical decision-support strategy for identifying biomarkers with greater translational potential in heterogeneous cancers such as CCA.

## 1. Introduction

Cholangiocarcinoma (CCA) is an aggressive malignancy of the biliary tract and represents the second most common primary liver cancer worldwide. Its incidence continues to rise globally, with a particularly high burden in Southeast Asia where liver fluke infection remains endemic [1]. Despite advances in systemic therapy, most patients are diagnosed at advanced stages, and the standard first-line regimen of gemcitabine–cisplatin provides only limited survival benefit, with a median overall survival of approximately 11–12 months [2]. These clinical challenges underscore the urgent need for improved strategies for early detection and biomarker-guided translational research in CCA.

A major obstacle to effective biomarker development in CCA is its pronounced biological heterogeneity. Extensive inter- and intra-tumoral diversity at genomic, epigenetic, and signaling levels has hindered the identification of molecular markers that remain robust across tumor subtypes and patient populations [3]. Conventional biomarker discovery approaches typically focus on identifying molecules that show statistically significant differences between tumor and normal samples within a single biological context—most commonly tumor tissue or a single biofluid such as blood. While such strategies can yield numerous candidate markers, many fail during clinical translation because they do not account for regulatory robustness, biological heterogeneity, or whether tumor-associated signals are consistently preserved in clinically accessible, non-invasive compartments.

Extracellular vesicles (EVs) have emerged as important mediators of intercellular communication and as potential carriers of disease-relevant molecular information. EVs are nanoscale, membrane-bound particles released by nearly all cell types and transfer proteins, lipids, and nucleic acids between cells [4]. In cancer, EVs actively contribute to multiple hallmarks of malignancy, including angiogenesis, metastasis, and immune modulation, through the dissemination of oncogenic and regulatory cargo [5,6]. Importantly, small EVs reflect regulated cellular processes rather than passive cellular debris, making them attractive substrates for biomarker discovery [7].

Recent advances in mass spectrometry-based proteomics and computational biology have enabled comprehensive profiling of EV cargo, facilitating systematic interrogation of disease-associated signaling networks. However, most EV-based biomarker studies remain confined to single sample types or emphasize subtype-enriched alterations, which may limit their applicability in heterogeneous clinical settings. Moreover, conventional EV studies often implicitly assume that tumor-associated signals are uniformly propagated into circulating biofluids, an assumption that is rarely examined explicitly at the discovery stage.

In contrast, the present study adopts a conceptually distinct, proof-of-concept strategy. Rather than prioritizing biomarkers solely based on differential expression within a single biological context, we implement an EV-guided, multi-compartment discovery framework that interrogates biomarker behavior across a hierarchy of biological compartments with increasing translational accessibility. By examining EV-associated molecular features derived from biologically distinct CCA cell models and integrating these findings with tissue-level molecular data and exploratory biofluid-derived EV analyses, this approach evaluates not only whether candidate biomarkers are tumor-associated, but also whether their signals demonstrate stability and regulatory coherence across different biological contexts.

Within this framework, tumor heterogeneity is leveraged as a stress test for biomarker robustness rather than treated as a confounding factor. The objective is not to maximize the number of candidate markers, but to prioritize those that exhibit consistent regulation across molecular layers and retain biologically interpretable signals in compartments with translational relevance. By integrating multivariate EV proteomic analysis with public transcriptomic, epigenetic, alternative promoter usage, copy-number, and miRNA regulatory datasets, this study establishes a discovery-stage framework that explicitly links molecular relevance with translational feasibility. Although applied here to CCA, this EV-guided, multi-compartment strategy is inherently generalizable and may be adapted to other heterogeneous malignancies where conventional biomarker discovery approaches face similar translational limitations. Together, this study establishes a discovery-stage framework that reframes biomarker identification as a compartment-aware decision-support process rather than a single-context screening exercise.

## 2. Materials and Methods

### 2.1. Cell Culture and Proliferation Assay

Four human cell lines were used in this study, including three CCA cell lines (CL-6, HuCCT-1, and HuH-28) and one immortalized normal cholangiocyte (MMNK-1). CL-6 was kindly provided by Associate Professor Adisak Wongkajornsilp (Mahidol University, Thailand), whereas HuCCT-1, HuH-28, and MMNK-1 were obtained from the Japanese Collection of Research Bioresources (JCRB, Japan). CL-6, HuCCT-1, and HuH-28 were maintained in RPMI-1640 medium, while MMNK-1 was cultured in Dulbecco’s Modified Eagle Medium (DMEM). All media were supplemented with 10% (*v*/*v*) heat-inactivated, EV-depleted fetal bovine serum and 100 IU/mL antibiotic–antimycotic. Cells were incubated at 37 °C in a humidified atmosphere containing 5% CO_2_ (HERAcell 150i, Thermo Scientific, Waltham, MA, USA).

Cell proliferation of CL-6, HuCCT-1, HuH-28, and MMNK-1 cells was evaluated using the MTT colorimetric assay. To functionally assess biological heterogeneity among the CCA cell models prior to EV isolation, antiproliferative sensitivity to cisplatin was evaluated. Cells were seeded in 96-well plates (1 × 10^4^ cells/well) and incubated overnight for attachment before treatment with cisplatin at serial concentrations (1.56–200 µg/mL) for 48 h under standard conditions (37 °C, 5% CO_2_). After treatment, 20 µL of MTT solution (5 mg/mL in PBS) was added and incubated for 4 h. Formazan crystals were dissolved in 100 µL DMSO, and absorbance was measured at 570 nm using a Varioskan Flash microplate reader (Thermo Fisher Scientific, Waltham, MA, USA). All experiments were performed in triplicate. IC_50_ values were calculated using CalcuSyn™ v2.11 (Biosoft, Cambridge, UK). The selectivity index (SI) was defined as the ratio of IC_50_ in normal MMNK-1 to IC_50_ in CCA cells.

### 2.2. Extracellular Vesicle (EV) Isolation

#### EV Isolation from Cell Culture Supernatant

EV-depleted fetal bovine serum (FBS) was prepared following MISEV2018-compliant protocols. Briefly, FBS (Gibco, NY, USA) was heat-inactivated at 56 °C for 30 min, sequentially centrifuged at 300× *g* for 10 min, 2000× *g* for 10 min, and 10,000× *g* for 30 min to remove cells and large vesicles. The supernatant was filtered through a 0.2 µm membrane (Millipore, Burlington, MA, USA) and ultracentrifuged at 100,000× *g* for 70 min at 4 °C (Beckman Coulter, Brea, CA, USA). The clarified supernatant was collected and stored at –20 °C until use. Depletion efficiency was confirmed by transmission electron microscopy (TEM). The resulting EV-free FBS was used for all cell culture, proliferation, and proteomic experiments.

EVs were isolated from conditioned media of CCA (CL-6, HuCCT-1, and HuH-28) and normal cholangiocyte (MMNK-1) cells following MISEV2018-compliant differential ultracentrifugation protocols [8]. Conditioned media were sequentially centrifuged at low speeds to remove cells and debris, filtered through a 0.22 µm membrane, and ultracentrifuged at 100,000× *g* for 70 min at 4 °C using an Optima™ L-100XP ultracentrifuge (Beckman Coulter, Brea, CA, USA) [9]. EV pellets were washed once with PBS, re-pelleted under identical conditions, and resuspended in 200 µL PBS. All EV preparations were stored at −80 °C until downstream analyses. To ensure reproducibility, the entire discovery workflow was performed in two independent biological replicates. This involved starting from separate cell culture passages for all four lines (CL-6, HuCCT-1, HuH-28, and MMNK-1), followed by independent EV isolation and purification procedures for each batch.

## 3. EV Isolation from Human Biofluids

Serum and urine samples were obtained from patients diagnosed with intrahepatic CCA (iCCA) and healthy controls at Sakon Nakhon Hospital, Sakon Nakhon, Thailand. Blood samples were allowed to clot at room temperature and centrifuged to obtain serum, while midstream urine samples were collected under sterile conditions. Aliquots were stored at −80 °C until EV isolation. Patients aged 18 years or older with advanced-stage iCCA were eligible for enrollment. The inclusion criteria were as follows: (i) confirmation of unresectable or metastatic iCCA by ultrasonography, contrast-enhanced computed tomography (CT), or magnetic resonance cholangiopancreatography (MRCP), with serum carbohydrate antigen 19-9 (CA19-9) levels > 129 U/mL; and (ii) the presence of at least one measurable tumor lesion with a longest diameter ≥ 20 mm according to the Response Evaluation Criteria in Solid Tumors (RECIST version 1.1). For the discovery phase, samples from 10 individuals per group were pooled (equal volume from each subject) prior to EV isolation to minimize inter-individual variability.

For serum-derived EVs, pooled serum samples were diluted 1:1 with sterile phosphate-buffered saline (PBS) and centrifuged at 2000× *g* for 30 min at 4 °C to remove cells and large debris. The supernatant was transferred and centrifuged at 12,000× g for 45 min at 4 °C to eliminate residual debris and large vesicles. The clarified supernatant was filtered through a 0.22 µm membrane and subsequently ultracentrifuged at 110,000× *g* for 2 h at 4 °C using an Optima™ L-100XP ultracentrifuge (Beckman Coulter, Brea, CA, USA) equipped with an SW41 Ti swinging-bucket rotor. The resulting pellets were washed with PBS and ultracentrifuged again at 110,000× *g* for 70 min at 4 °C. Finally, EV pellets were resuspended in 200 µL PBS and stored at −80 °C until downstream analyses [9].

For urinary EV isolation, pooled urine samples were isolated by differential ultracentrifugation adapted from Puhka et al., 2017 [10], with minor modifications. Urine samples were thawed at 37 °C and vortexed before centrifugation at 2000× *g* for 30 min and subsequently at 17,000× *g* for 45 min to remove debris and protein aggregates. The supernatant was filtered through a 0.22 µm membrane and ultracentrifuged at 110,000× *g* for 90 min using an Optima™ L-100XP ultracentrifuge (Beckman Coulter, Brea, CA, USA) equipped with an SW41 Ti swinging-bucket rotor. The pellet was washed with PBS and ultracentrifuged again at 110,000× *g* for 70 min. Finally, EVs were resuspended in 200 µL PBS and stored at −80 °C until further analysis.

### 3.1. Characterization of Extracellular Vesicles (EVs)

The morphology and size distribution of EVs were evaluated using transmission electron microscopy (TEM) and nanoparticle tracking analysis (NTA) in accordance with MISEV2018 guidelines [8]. For TEM, EVs isolated from EV-depleted FBS and from the conditioned media of CL-6, HuCCT-1, HuH-28, and MMNK-1 cells were adsorbed onto Formvar-coated copper grids, negatively stained with 1% uranyl acetate, and imaged at 100 kV to confirm their characteristic cup-shaped morphology (30–150 nm). For NTA, EVs derived from CL-6 cells, pooled CCA serum, and pooled CCA urine were analyzed to confirm particle size distribution and concentration, using a multispectral tracking system (ViewSizer 3000, HORIBA Scientific, Kyoto, Japan). EV suspensions were diluted in 0.1 µm-filtered PBS to obtain 20–120 particles per frame, and mean values were averaged from 25 videos recorded at 25 ± 1 °C. Particle size parameters and concentration (particles/mL) were calculated using the ViewSizer software (Version 2.13).

NTA was performed primarily for quality control and confirmation of small EV size distribution across representative cellular and biofluid-derived samples, rather than for quantitative comparison between conditions or sample types.

### 3.2. Database Cross-Validation of EV Proteins

Candidate EV proteins were cross-checked against the ExoCarta database (accessed on 1 December 2025) using human UniProt IDs. For each protein, we verified whether it has been previously reported in human extracellular vesicles and recorded the type of experimental evidence available (e.g., MS, WB, IF). Database evidence was used only as external support for EV association, rather than direct validation of our isolates.

### 3.3. Proteomic Analysis

EV pellets were lysed in 0.5% sodium dodecyl sulfate (SDS) and subjected to cold acetone precipitation at −20 °C for 1 h. After centrifugation at 13,000× *g* for 15 min, protein pellets were resuspended in 0.5% SDS. Protein concentrations were quantified using the Pierce™ BCA Protein Assay Kit (Thermo Fisher Scientific, USA) following the manufacturer’s instructions. Absorbance was measured at 562 nm, and protein concentrations were calculated from a bovine serum albumin (BSA) standard curve. Samples were normalized to 5 µg of total protein per injection for LC–MS/MS analysis.

For each cell model, three technical LC–MS/MS replicates were performed. These replicates represented repeated injections derived from the same EV isolation and protein extraction and were included to assess analytical reproducibility and instrument stability. No independent biological EV isolations were performed for proteomic analysis. LC–MS/MS acquisition for all EV samples was conducted within a single analytical batch on the same instrument platform under consistent operating conditions to minimize inter-batch variability and instrument drift.

For protein digestion, 5 µg were treated with buffer I (10 mM each of dithiothreitol and ammonium bicarbonate) for 1 h, followed by buffer II (100 mM iodoacetamide and 10 mM ammonium bicarbonate) for 1 h. The extracted proteins were mixed with buffer I to terminate the iodoacetamide reaction and digested with trypsin (10 ng trypsin in 50% acetonitrile and 10 mM ammonium bicarbonate). The samples were incubated overnight, and protein identification was performed using LC-MS/MS. The digested proteins were injected onto a µ-pre-column (Monolithic Trap Column, 200 µm i.d. × 5 mm) coupled with the Ultimate 3000 LC system (Dionex, Thermo Fisher Scientific, Waltham, MA, USA), and ESI-Ion Trap MS (HCT ultra PTM Discovery System, Bruker Daltonik, Bremen, Germany) with electrospray at a flow rate of 20 µL/min. Proteins were separated on a nano-column (Monolithic Nano Column, 100 µm i.d.× 5 cm) (Thermo Fisher Scientific, Amsterdam, The Netherland) with a solvent gradient mobile phase consisting of solvent A (0.1% formic acid) and solvent B (50% water, 50% acetonitrile, and 0.1% formic acid), running at a flow rate of 1 µL/min for 20 min.

The DecyderMS software (version 2.0) was utilized to quantify peptides in individual samples derived from MS/MS data, as described by Johansson et al. [11] and Thorsell et al. [12]. In summary, the raw LC-MS/MS data files were imported into the software and processed within the Pepdetect module of DecyderMS. Ion counts for each peptide were integrated, and the log2 transformation of these values was reported. The resultant data were then uploaded to the Pepmatch module, where peptides were matched based on their *m*/*z* ratios and retention times. The integrated peptide counts formed the basis for statistical analyses to evaluate the probability that observed changes were statistically significant. Additionally, false discovery rates were calculated from the data.

MASCOT software (version 2.2) was employed to correlate MS/MS spectra obtained from DecyderMS software with the *Homo sapiens* protein database (downloaded on 26 June 2022). The search criteria included the following parameters: taxonomy (*Homo sapiens*), enzyme (trypsin), variable modifications (oxidation of methionine residues), mass values (monoisotopic), protein mass (unrestricted), peptide mass tolerance (1.0 Da), fragment mass tolerance (±0.4 Da), peptide charge states (1+, 2+, and 3+), and a maximum number of missed cleavages [13]. Proteins were identified using one or more peptides with an individual MASCOT score corresponding to *p* < 0.05 and were subsequently annotated with UniProtKB/Swiss-Prot entries “http://www.uniprot.org/ (accessed on 1 October 2025)”.

### 3.4. Statistical and Integrative Bioinformatic Analyses

Differential proteomic analyses were conducted to examine molecular heterogeneity among CCA models and to identify cancer-associated EV proteins. EV-enriched proteomes derived from three CCA cell lines (CL-6, HuCCT-1, and HuH-28) were first analyzed using partial least squares–discriminant analysis (PLS-DA) to evaluate intrinsic proteomic differences among CCA models. Subsequently, EV proteomes from each CCA cell line were compared with those from the normal cholangiocyte (MMNK-1) to identify cancer-associated protein alterations. Normalized protein intensity values were processed using MetaboAnalyst 6.0 (https://www.metaboanalyst.ca/; accessed on 15 October 2025). Prior to statistical analysis, missing values were replaced with zero under the assumption that undetected proteins reflected signals below the LC–MS/MS detection limit. This procedure was applied consistently across all comparison groups. Proteins with a false discovery rate (FDR)-adjusted *p*-value < 0.05 and an absolute fold change ≥ 2 were considered significantly altered. Volcano plots were used to visualize differential expression patterns, while proteins with variable importance in projection (VIP) scores ≥ 2 in the PLS-DA models were considered influential and prioritized for downstream analyses. Proteins identified by both volcano analysis and PLS-DA were subsequently integrated using Venn diagram analysis to determine shared EV-associated proteins across all three CCA models, as well as proteins uniquely altered in individual CCA cell lines relative to normal cholangiocytes.

Prioritized proteins were subsequently subjected to integrative bioinformatic analyses to explore their biological relevance, regulatory mechanisms, and translational potential.

Functional annotation and pathway enrichment analyses were performed using the PANTHER database “http://www.pantherdb.org (accessed on 15 October 2025)”. In silico validation of corresponding gene expression was conducted using GEPIA, integrating TCGA-CHOL and GTEx datasets “http://gepia.cancer-pku.cn (accessed on 15 October 2025)”, to enable comparison between tumor and normal bile duct tissues. Protein localization, tissue specificity, and detectability in blood were assessed using the Human Protein Atlas “https://www.proteinatlas.org (accessed on 15 October 2025)”. Genomic and epigenetic features—including DNA methylation status, copy number alterations, and mutation frequency—were evaluated using UALCAN “http://ualcan.path.uab.edu (accessed on 15 October 2025)” and UCSC Xena “https://xenabrowser.net (accessed on 15 October 2025) ”, respectively.

Protein–protein interaction (PPI) networks were constructed using STRING “https://string-db.org (accessed on 15 October 2025)”, restricted to high-confidence, experimentally supported interactions (interaction score > 0.7), to identify potential hub-associated proteins and shared regulatory contexts.

To evaluate the diagnostic relevance of prioritized candidates at the tissue level, receiver operating characteristic (ROC) curve analysis was performed using normalized RNA-seq expression data derived from TCGA and GTEx cohorts. Area under the curve (AUC) values were calculated to assess the ability of candidate genes to discriminate CCA tumors from normal bile duct tissues.

To further explore the relationship between candidate gene expression and the tumor immune microenvironment, immune cell infiltration was estimated using the TIMER2.0 algorithm “https://timer.cistrome.org (accessed on 15 October 2025)”. Correlations between gene expression levels and inferred immune cell populations were analyzed to provide contextual insight into tumor-associated immune landscapes. 

Potential post-transcriptional regulation was investigated using the miRTarget database “https://www.mirtarget.com/ (accessed on 15 October 2025)”. Candidate miRNAs were selected based on the following criteria:

(1) altered mRNA expression following ectopic miRNA introduction;

(2) altered mRNA expression upon miRNA knockout or knockdown;

(3) predicted miRNA–target pairing supported by at least five independent algorithms; and

(4) a negative correlation with mRNA expression (correlation coefficient ≤ −0.1) across TCGA datasets.

## 4. Results

### 4.1. Extracellular Vesicle (EV) Morphology and Size Characterization

**Figure 1:**
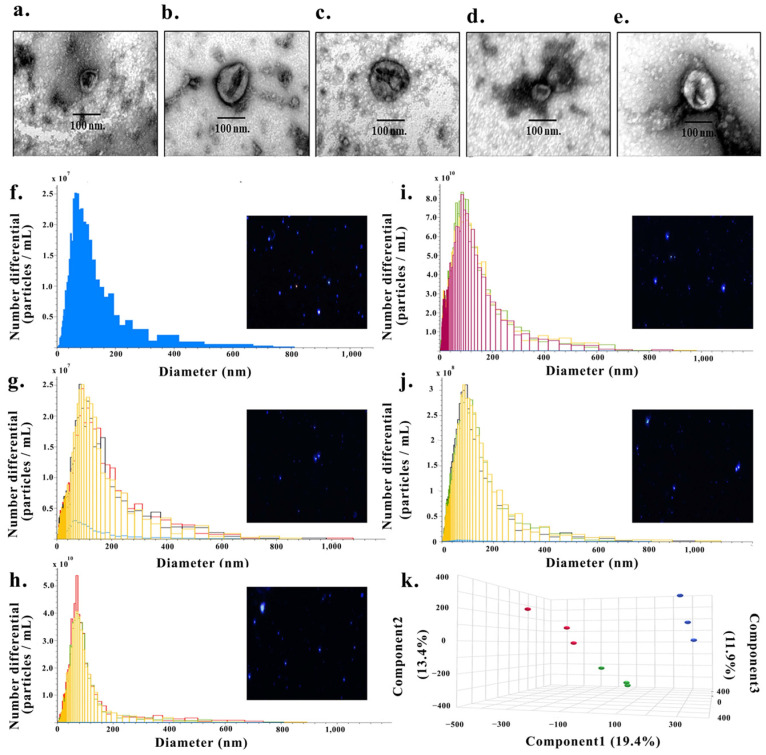
Characterization of extracellular vesicles (EVs) derived from CCA cell lines and controls. Transmission electron microscopy (TEM) images showing the morphology of exosome-like vesicles isolated from fetal bovine serum (FBS) (**a**), and the conditioned media of CL-6 (**b**), HuCCT-1, (**c**) HuH-28 (**d**), and MMNK-1 cells (**e**). Typical round or cup-shaped vesicles with diameters ranging from 30 to 150 nm were observed. Nanoparticle tracking analysis (NTA) profiles demonstrating the size distribution and concentration of EVs: PBS served as a blank control (**f**), culture media containing EV-depleted FBS as a negative control (**g**), EVs isolated from CL-6 conditioned medium (**h**), serum-derived EVs (**i**), and urine-derived EVs (**j**). Different colored lines represent three independent measurements performed for each sample. NTA confirmed particle populations within the expected size range for EVs. NTA confirmed particle populations within the expected size range for extracellular vesicles (EVs). Partial least squares–discriminant analysis (PLS-DA) of EV proteomic profiles derived from three CCA cell lines (CL-6, HuCCT-1, and HuH-28) (**k**). In panel (**k**), red dots represent CL-6, green dots represent HuCCT-1, and blue dots represent HuH-28.

Transmission electron microscopy (TEM) revealed that EVs isolated from FBS and from the culture media of CL-6, HuCCT-1, HuH-28, and MMNK-1 cells exhibited the characteristic round to cup-shaped morphology (Figure 1a–e)**.** The vesicles had mean diameters of approximately 70 nm for FBS-derived, 100 nm for CL-6 and HuCCT-1, 80 nm for HuH-28, and 100 nm for MMNK-1, consistent with the expected exosomal size range (30–150 nm) (Figure 1a–e)**.**

**Figure 2:**
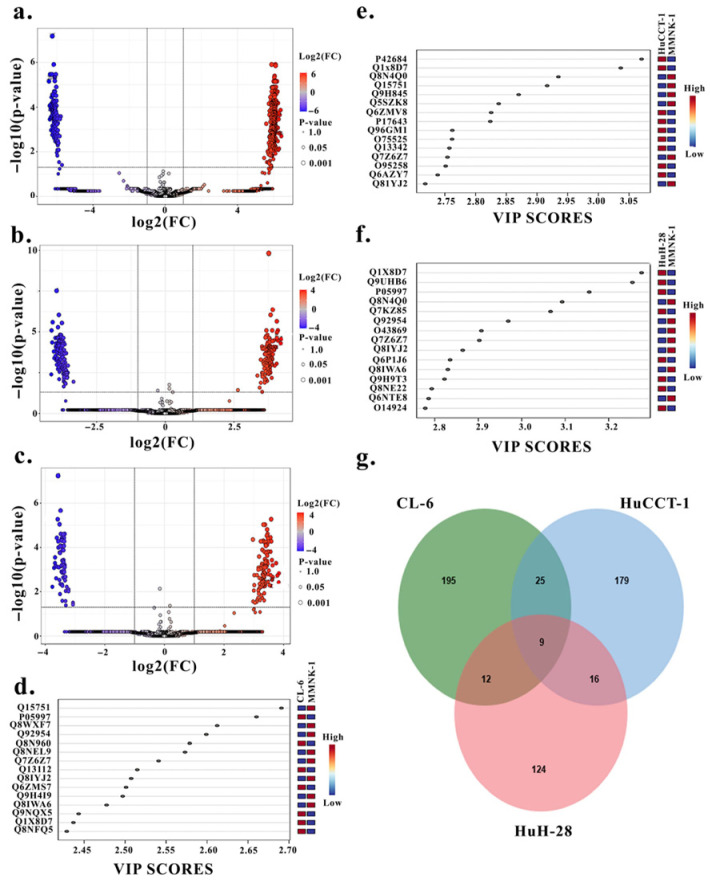
Differential EV proteome profiling, feature selection, overlap, and in silico Biomarker validation. Volcano plots comparing EV protein abundances in each CCA cell line with the normal cholangiocyte (MMNK-1): CL-6 vs. MMNK-1 (**a**), HuCCT-1 vs. MMNK-1 (**b**), and HuH-28 vs. MMNK-1 (**c**). PLS-DA variable importance-in-projection (VIP) plots highlighting discriminative EV proteins for each cancer/normal comparison: CL-6 vs. MMNK-1 (**d**), HuCCT-1 vs. MMNK-1 (**e**), and HuH-28 vs. MMNK-1 (**f**). Venn diagram showing the overlap of proteins jointly retained by both differential expression (Volcano) and feature-selection (PLS-DA) criteria across all three CCA cell lines; the triple intersection identifies nine shared EV-associated protein candidates (**g**).

**Figure 3:**
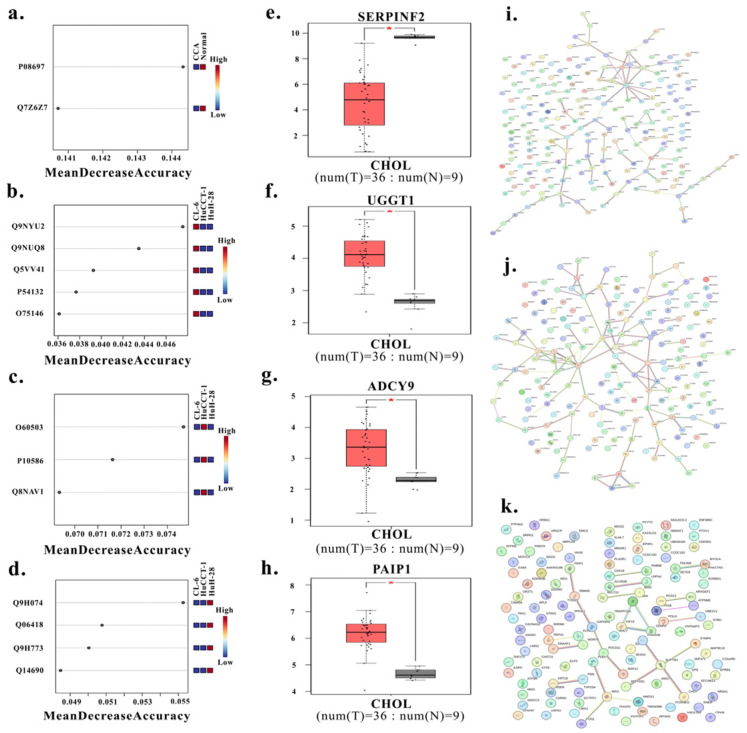
Integrated biomarker prioritization and subtype-specific hub-network mapping. Random-forest feature selection of EV-derived proteins identifying: a pan-CCA biomarker candidate (SERPINF2) (**a**), CL-6-associated candidate biomarker linked to an angiogenic dominant subtype (UGGT1) (**b**), HuCCT-1-associated candidate biomarker linked to a cytokine/inflammation-dominant subtype (ADCY9) (**c**), and HuH-28-associated candidate biomarker linked to a G-protein-related subtype (PAIP1) (**d**). Validation of differential gene expression in CCA tumor vs. normal tissues using GEPIA for SERPINF2 (pan-CCA candidate) (**e**), UGGT1 (angiogenic-dominant CL-6 subtype candidate) (**f**), ADCY9 (inflammation dominant HuCCT-1 subtype candidate) (**g**), and PAIP1 (G-protein-associated HuH-28 subtype candidate) (**h**). STRING protein–protein interaction networks showing hub-node organization within subtype-associated EV proteomes from CL-6 (angiogenic-dominant) (**i**), HuCCT-1 (cytokine/inflammation-dominant) (**j**), and HuH-28 (G-protein dominant) cells (**k**). * indicate statistically significant differences between tumor and normal tissues (*p* < 0.01).

**Figure 4:**
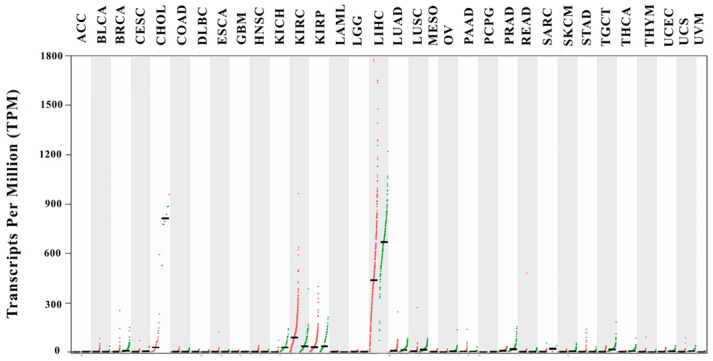
Shows the expression levels of SERPINF2 across multiple cancer types using RNA-sequencing data from TCGA tumor samples and matched normal tissues. Gene expression is presented as transcripts per million (TPM), with individual dots representing individual samples. Tumor samples are indicated in red, whereas normal tissues are shown in green. Cancer types are labeled along the x-axis according to TCGA nomenclature. The black line within each box represents the median expression level.

**Figure 5:**
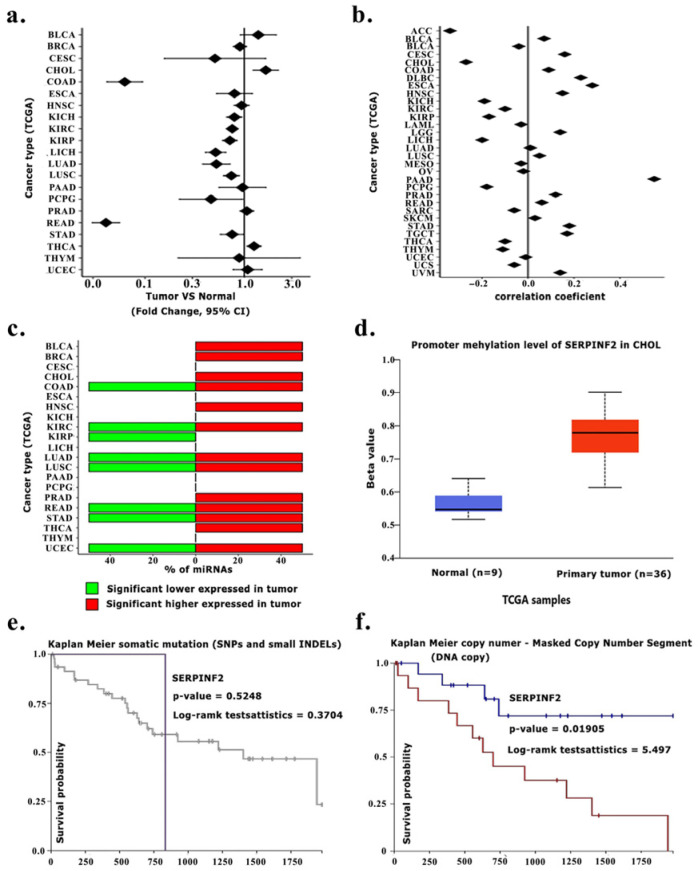
Multi-omics and clinical relevance of SERPINF2 and its regulation by miR-125a. Differential expression of miR-125a between tumor and normal tissues across multiple TCGA cancer types (**a**). Correlation analysis between miR-125a and SERPINF2 expression in diverse cancers, showing inverse associations in several tumor types (**b**). Distribution of miR-125a upregulation (red) or downregulation (green) across TCGA datasets (**c**). Promoter methylation levels of SERPINF2 in CCA compared with normal tissues, indicating hypermethylation in tumors (**d**). Kaplan–Meier survival analysis of patients stratified by SERPINF2 somatic mutation status (SNPs and small INDELs) (**e**). Kaplan–Meier survival analysis based on SERPINF2 copy number variation (DNA copy segment), demonstrating prognostic impact in TCGA cohorts (**f**).

Nanoparticle tracking analysis (NTA) further confirmed the presence and size distribution of small EVs. Five particle populations were analyzed using a ViewSizer 3000 system, including: (1) blank 0.1 µm-filtered PBS (single measurement) (Figure 1f), (2) EV-depleted culture medium (*n* = 3) (Figure 1g), (3) CL-6 EVs (*n* = 3) (Figure 1h), (4) CCA serum EVs pooled from 10 patients (*n* = 3) (Figure 1i), and (5) CCA urine EVs pooled from 10 patients (*n* = 3) (Figure 1j). The blank showed minimal background noise and was used for background subtraction. After correction, CL-6 EVs exhibited a mean particle concentration of 1.29 ± 0.31 × 10^11^ particles/mL with a median diameter of 70.26 nm (70.11–71.52 nm), confirming a predominant small-EV population. CCA serum EVs showed a mean concentration of 6.78 ± 0.38 × 10^8^ particles/mL with a median diameter of 85.6 nm (84.07–93.64 nm), while CCA urine EVs demonstrated a detectable EV population with a mean concentration of 1.32 ± 0.12 × 10^11^ particles/mL and a median diameter of 84.93 nm (77.05–95.44 nm), all consistent with characteristic small EV sizes. These analyses were performed solely to confirm successful EV isolation and were not intended for comparative quantitative interpretation among the sample types.

### 4.2. Antiproliferative Activity of Cisplatin in CCA Cell Lines

The antiproliferative effect of cisplatin was evaluated in three CCA cell lines (CL-6, HuCCT-1, and HuH-28) and the normal cholangiocyte line MMNK-1 using the MTT assay. IC_50_ values are summarized in Table 1. Cisplatin exhibited strong antiproliferative activity in CL-6 cells, with a median IC_50_ value of 5.0 µg/mL (range: 4.5–5.37 µg/mL). HuCCT-1 cells showed moderate sensitivity to cisplatin, with an IC_50_ value of 8.0 µg/mL (range: 7.46–9.1 µg/mL). In contrast, HuH-28 cells demonstrated markedly reduced sensitivity, with a substantially higher IC_50_ value of 55.0 µg/mL (range: 51.0–57.9 µg/mL). The normal cholangiocyte line MMNK-1 exhibited lower IC_50_ values compared with CL-6 and HuCCT-1 cells, allowing calculation of selectivity indices that varied among the CCA models. Overall, cisplatin displayed distinct response profiles across the three CCA cell lines. These differential cisplatin sensitivities reflect intrinsic biological heterogeneity among the cell models used in this study.

### 4.3. PLS-DA Reveals Intrinsic Proteomic Heterogeneity Among CCA-Derived EVs

To determine whether the observed functional differences were reflected at the molecular level, EV-enriched proteomic profiles from CL-6, HuCCT-1, and HuH-28 cell lines were analyzed using partial least squares–discriminant analysis (PLS-DA). The PLS-DA score plot demonstrated clear separation among the three CCA models, with CL-6, HuCCT-1, and HuH-28 forming distinct and non-overlapping clusters. Replicate EV-enriched proteomic preparations from each cell line clustered tightly, indicating good analytical reproducibility of the EV proteomic data. Separation was observed across multiple components, with Component 1, Component 2, and Component 3 explaining 19.4%, 13.4%, and 11.9% of the variance, respectively (Figure 1k). Together, these results indicate pronounced intrinsic molecular heterogeneity among EV proteomes derived from different CCA cell lines, independent of formal subtype classification.

Taken together, cisplatin sensitivity assays and PLS-DA-based proteomic profiling consistently demonstrate functional and molecular heterogeneity among the three CCA cell lines, providing a rationale for subsequent identification of EV-associated proteins that are conserved across heterogeneous CCA models.

## 5. Candidate Biomarker Identification and Regulatory Mechanisms

### 5.1. Differential EV Proteomic Profiling Reveals Shared and Subtype-Specific Signatures Across CCA Cell Lines

Differential expression analyses between each CCA cell line (CL-6, HuCCT-1, HuH-28) and the normal cholangiocyte MMNK-1 revealed hundreds of significantly altered exosomal proteins. Volcano plots (FDR < 0.05, fold change ≥ 2) combined with partial least squares–discriminant analysis (PLS-DA; VIP ≥ 2.0) identified 241 proteins in CL-6 vs. MMNK-1, 229 in HuCCT-1 vs. MMNK-1, and 161 in HuH-28 vs. MMNK-1 (Figure 2a–f). Integration of the three datasets using Venn diagrams revealed nine proteins consistently shared across all CCA lines, alongside 195, 179, and 124 proteins uniquely altered in CL-6, HuCCT-1, and HuH-28, respectively (Figure 2g). Functional enrichment analysis using PANTHER highlighted distinct but biologically relevant pathway signatures across models, including an angiogenesis-dominant subtype (CL-6), a cytokine/inflammation-dominant subtype (HuCCT-1), and a G-protein-signaling-dominant subtype (HuH-28), underscoring intrinsic molecular heterogeneity among CCA models.

### 5.2. Integrative Prioritization Identifies SERPINF2 as a Conserved EV-Associated Candidate Biomarker

Candidate prioritization was performed by integrating GEPIA-based gene expression analysis (*p* < 0.01, |log2FC| ≥ 1), Human Protein Atlas annotation (tissue specificity and blood detectability), random forest feature ranking, and ExoCarta annotation. This multi-criteria approach consistently identified SERPINF2 (P08697) as the top candidate biomarker deregulated across all three CCA EV proteomes (Figure 3a–h). In contrast, UDP-glucose:glycoprotein glucosyltransferase 1 (UGGT1; Q9NYU2), adenylate cyclase type 9 (ADCY9; O60503), and polyadenylate-binding protein–interacting protein 1 (PAIP1; Q9H074) emerged as subtype-specific markers associated with CL-6, HuCCT-1, and HuH-28, respectively (Figure 3i–k).

Pan-cancer expression analysis further demonstrated that SERPINF2 downregulation was particularly pronounced in CCA compared with matched normal tissues, whereas tumor–normal differences were less distinct in most other cancer types, highlighting the relative specificity of SERPINF2 loss in CCA (Figure 4).

### 5.3. Post-Transcriptional Regulation of SERPINF2 via miRNA-Mediated Suppression

To explore post-transcriptional regulation, SERPINF2 was further analyzed using the miRTarget platform. Expression analysis revealed that miR-125a was significantly upregulated in CHOL tumors compared with normal tissues (Figure 5a), and correlation analysis demonstrated a negative association between miR-125a and SERPINF2 expression (Figure 5b). In addition, SERPINF2-targeting miRNAs, including miR-125a, were enriched in tumor samples across multiple cancer types (Figure 5c). Together, these findings support a potential contribution of miRNA-mediated post-transcriptional regulation to SERPINF2 downregulation in CCA.

### 5.4. Genomic and Epigenetic Features Associated with SERPINF2 Downregulation

**Figure 6:**
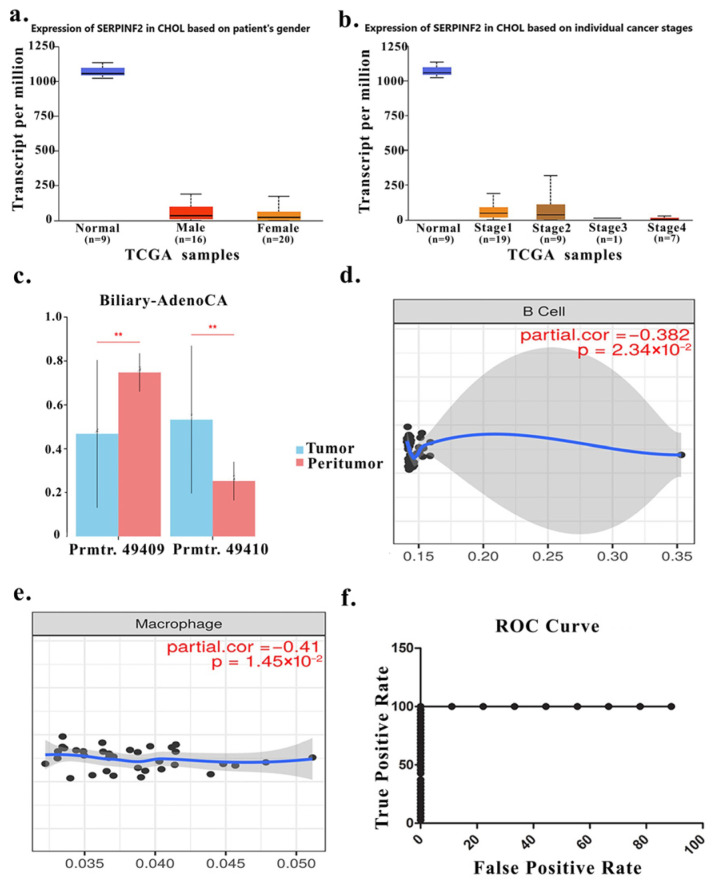
Multi-layer characterization of SERPINF2 expression, regulation, immune association, and diagnostic performance in CCA. Box plots showing SERPINF2 expression levels (transcripts per million, TPM) in CHOL tumors stratified by patient sex using TCGA data. No significant sex-dependent differences were observed, with consistently reduced expression in tumor samples compared with normal bile duct tissues (**a**). SERPINF2 expression stratified by individual tumor stages (Stage I–IV) in CHOL. Reduced expression was observed across all stages, indicating stage-independent downregulation (**b**). Promoter methylation analysis of SERPINF2 in biliary adenocarcinoma, comparing tumor and peritumoral tissues. Differential activity of two promoter regions (Prmtr_49409 and Prmtr_49410) suggests context-dependent promoter usage and epigenetic regulation (**c**). Partial correlation analysis between SERPINF2 expression and estimated B-cell infiltration, demonstrating a significant inverse association (**d**). Partial correlation analysis between SERPINF2 expression and macrophage infiltration, also revealing a significant negative association (**e**). Black dots represent individual tumor samples. The blue line indicates the fitted regression (smoothed) trend line. The gray shaded area represents the 95% confidence interval of the regression model. Both analyses demonstrate significant inverse associations. Receiver operating characteristic (ROC) curve evaluating the ability of SERPINF2 expression to discriminate CHOL tumor tissues from normal bile duct tissues based on TCGA and GTEx datasets. The curve demonstrates strong diagnostic performance at the tissue level (**f**). ** indicate statistically significant differences between groups (*p* < 0.01).

Beyond post-transcriptional regulation, genomic and epigenetic alterations affecting SERPINF2 were further examined. Promoter methylation levels were significantly higher in CHOL tumors compared with normal tissues (β ≈ 0.75 vs. 0.56), consistent with the observed reduction in SERPINF2 expression (Figure 5d). Stratified analyses indicated that somatic mutations in SERPINF2 were rare and showed no association with patient survival (Figure 5e). In contrast, copy number alterations demonstrated a significant association with overall survival, with gene copy loss correlating with poorer prognosis (*p* = 0.019; Figure 5f). Notably, SERPINF2 expression did not differ significantly by patient sex (Figure 6a) or tumor stage (Figure 6b), with consistently reduced expression observed across both early and advanced disease stages.

To further investigate transcriptional mechanisms underlying SERPINF2 downregulation, alternative promoter usage was analyzed using TCGA-CHOL (Biliary-AdenoCA) data. Two distinct SERPINF2 promoters (prmtr.49409 and prmtr.49410) located on chromosome 17 were identified as transcriptionally active in biliary adenocarcinoma samples. Promoter activity analysis revealed significant differences between tumor and peritumoral tissues for both promoters. Specifically, prmtr.49409 showed significantly reduced activity in tumor samples compared with peritumoral tissues (mean activity 0.47 vs. 0.75, *p* = 0.0037), whereas prmtr.49410 exhibited higher activity in tumor tissues relative to peritumoral counterparts (mean activity 0.53 vs. 0.25, *p* = 0.0037) (Figure 6c).

### 5.5. Association Between SERPINF2 Expression and Tumor Immune Contexture

To contextualize SERPINF2 expression within the tumor microenvironment, immune infiltration analysis was performed using TIMER2.0. SERPINF2 expression demonstrated moderate inverse correlations with estimated B cell and macrophage infiltration levels in CHOL tumors (Figure 6d,e), suggesting that reduced SERPINF2 expression is associated with immune-enriched tumor contexts as inferred by in silico immune deconvolution.

### 5.6. Tissue-Level Discriminatory Performance of SERPINF2 Expression

Receiver operating characteristic (ROC) curve analysis using normalized RNA-seq data from TCGA-CHOL tumors and GTEx normal bile duct tissues demonstrated clear separation between tumor and normal samples. SERPINF2 expression achieved an area under the curve (AUC) of 1.00 (95% confidence interval: 1.00–1.00; *p* < 0.0001), indicating strong discriminatory performance at the tissue level (Figure 6f). These findings support the utility of SERPINF2 as a tissue-level candidate biomarker in a discovery-stage context.

### 5.7. Exploratory Characterization of Pooled Serum- and Urine-Derived EV Proteomes

**Figure 7:**
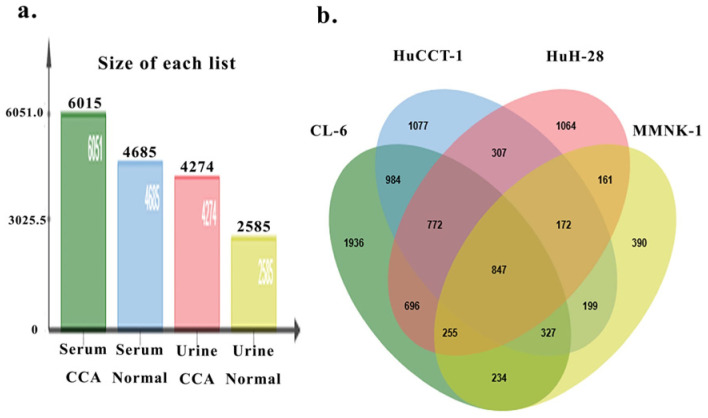
**Overview of EV proteome composition across biofluids and CCA cell models.** Bar chart showing the number of identified EV-associated proteins detected in serum- and urine-derived EVs from cholangiocarcinoma (CCA) patients and healthy controls (**a**). Serum EVs from CCA patients exhibited the highest number of detected proteins, followed by serum EVs from healthy controls, urine EVs from CCA patients, and urine EVs from healthy individuals, highlighting marked differences in protein complexity across biofluids and disease states. Venn diagram illustrating the overlap of EV-associated proteins identified from three CCA cell lines (CL-6, HuCCT-1, and HuH-28) and a normal cholangiocyte cell line (MMNK-1) (**b**). Both shared and cell line-specific protein subsets were observed, reflecting substantial inter-tumoral heterogeneity. The intersection highlights a core set of conserved EV proteins across malignant and non-malignant models, which were prioritized for downstream integrative and translational analyses.

Proteomic profiling of pooled extracellular vesicles (EVs) derived from human serum and urine revealed distinct global protein landscapes between CCA patients and healthy controls. In serum-derived EVs, a total of 6051 proteins were identified in CCA samples, compared with 4685 proteins in normal serum EVs, representing an approximate 1.29-fold increase in protein diversity in CCA (Figure 7a). Similarly, urine-derived EVs from CCA patients contained 4274 proteins, whereas 2585 proteins were detected in urine EVs from healthy individuals, corresponding to an approximate 1.65-fold increase in the number of identified proteins in CCA urine samples (Figure 7a).

To further delineate disease-associated EV protein signatures, comparative Venn diagram analyses were performed. In serum-derived EVs, comparison between CCA and normal samples identified a total of 7806 unique proteins, of which 3121 proteins (40.0%) were detected exclusively in CCA serum EVs, while 1755 proteins (22.0%) were detected exclusively in normal serum EVs. The remaining proteins were shared between the two groups.

In urine-derived EVs, comparison between CCA and normal samples revealed 5424 total proteins, with 2839 proteins (52.3%) uniquely detected in CCA urine EVs and 1150 proteins (21.2%) uniquely detected in normal urine EVs. These findings indicate a higher proportion of disease-specific proteins in urine-derived EVs compared with serum-derived EVs.

Integration of EV proteomic data from all four sample groups (CCA serum, normal serum, CCA urine, and normal urine) using a Venn diagram identified 847 proteins that were consistently detected across all biofluid conditions (Figure 7b). In contrast, SERPINF2 was localized within a subset of 390 proteins uniquely detected in urine-derived EVs from healthy individuals, and was absent from CCA urine EVs as well as from serum-derived EVs of both CCA patients and controls.

To characterize the biological functions associated with this urine-normal–specific protein subset, pathway enrichment analysis was performed using the PANTHER classification system. The 390-protein set was enriched across 71 biological pathways, with the inflammation mediated by chemokine and cytokine signaling pathway representing the most significantly populated pathway, involving seven associated proteins. Notably, SERPINF2 was assigned to the blood coagulation pathway, together with F2RL3, highlighting a functional association between SERPINF2 depletion and coagulation-related signaling in urine-derived EVs.

## 6. Discussion

### 6.1. An EV-Guided Proof-of-Concept Framework for Biomarker Prioritization in Heterogeneous CCA

CCA is characterized by marked inter- and intra-tumoral heterogeneity, which remains a central challenge for biomarker discovery and clinical translation. Numerous candidate biomarkers demonstrate statistically significant differences in specific cohorts yet fail to generalize across tumor subtypes, biological contexts, or clinically accessible sample types. These limitations highlight a broader, long-standing problem in biomarker research: despite an abundance of discovery-stage candidates reported across cancer types, only a small fraction successfully translate into clinical use.

While this high attrition rate is often attributed to biological complexity, cohort variability, or insufficient downstream validation, a critical limitation frequently arises much earlier in the biomarker development pipeline. Specifically, most discovery studies lack explicit decision criteria to determine which candidates should advance beyond initial identification. Candidate selection is therefore commonly driven by statistical significance within isolated biological contexts, implicitly assuming that such signals will remain robust across tumor heterogeneity and be preserved in clinically accessible compartments. This assumption-driven progression allows many biologically fragile or context-dependent markers to enter costly validation stages, contributing substantially to downstream translational failure.

To translate this conceptual shift into an operational workflow, we designed an EV-guided discovery framework that explicitly embeds prioritization criteria at the earliest stages of biomarker identification. The framework presented in this study directly addresses this structural gap by repositioning biomarker discovery as a decision-support process rather than a purely descriptive exercise. By introducing an explicit prioritization layer at the discovery stage, translational robustness is treated as a measurable and testable property rather than a post hoc outcome. Specifically, the framework (i) applies EV-guided profiling to enrich for molecular signals that are actively and selectively exported from tumor cells, thereby filtering out candidates unlikely to reach extracellular or biofluid compartments; (ii) leverages biological heterogeneity as a stress test, prioritizing candidates that exhibit conserved behavior across biologically distinct tumor models rather than subtype-restricted alterations; and (iii) evaluates compartment-dependent signal preservation early in the pipeline to inform biofluid suitability prior to large-scale clinical validation.

In this proof-of-concept study, we implemented this EV-guided, multi-layer integrative framework to prioritize molecular features conserved across biologically distinct CCA models. Rather than focusing on subtype-enriched or context-specific alterations, this approach exploits tumor heterogeneity as an analytical filter to identify EV-associated signals that persist despite underlying molecular diversity. This conceptual shift aligns with emerging perspectives in precision oncology, which emphasize robustness and signal stability over maximal candidate enumeration [14,15].

The clear separation of EV proteomic profiles among CL-6, HuCCT-1, and HuH-28 cell lines reinforces the intrinsic molecular diversity of CCA, consistent with prior transcriptomic and genomic classifications [16,17]. Importantly, the identification of a limited subset of EV-associated proteins shared across all models supports the concept that EV cargo may capture convergent disease-associated processes that persist across heterogeneous tumor states. From a proof-of-concept perspective, this observation demonstrates that EVs can function as a biologically informed molecular filter, enriching for candidates with greater resilience to heterogeneity. This is consistent with accumulating evidence that EVs selectively package functionally relevant cargo rather than representing passive cellular debris [7,18].

### 6.2. SERPINF2 as a Model Candidate Illustrating Multi-Layer Regulatory Robustness

Among conserved EV-associated candidates, SERPINF2 emerged as the most consistently deregulated protein across proteomic, transcriptomic, and regulatory layers. SERPINF2 (α2-antiplasmin) is a central regulator of fibrinolysis with established roles in fibrin stability, extracellular matrix remodeling, and vascular homeostasis [19]. While dysregulation of coagulation and fibrinolytic pathways has been implicated in cancer progression, angiogenesis, and immune modulation [20,21], SERPINF2 itself has received limited attention in CCA.

A key finding of this study is that SERPINF2 downregulation appears to be governed by coordinated multi-layer regulation rather than a single molecular event. Promoter hypermethylation likely contributes to transcriptional repression, consistent with widespread epigenetic alterations reported in CCA [17,22]. In parallel, post-transcriptional suppression mediated by miR-125a provides an additional regulatory axis, aligning with reports of miR-125a as a context-dependent modulator of tumor progression, angiogenesis, and immune signaling [23,24]. Evidence of alternative promoter usage further suggests that SERPINF2 expression may be shaped by context-dependent transcriptional architecture, a mechanism increasingly recognized in cancer biology [25,26].

From a proof-of-concept standpoint, this convergence across regulatory layers strengthens confidence that SERPINF2 loss reflects a biologically programmed and stable event rather than stochastic variation. Notably, SERPINF2 expression was independent of patient sex and tumor stage, supporting its potential relevance across heterogeneous clinical populations and reinforcing its suitability as a model candidate within this framework.

### 6.3. Immune Contextualization as an Added Layer of Biological Plausibility

Immune deconvolution analyses revealed an inverse association between SERPINF2 expression and estimated B-cell and macrophage infiltration. Although these observations are derived from in silico analyses and cannot establish causality, they are consistent with emerging evidence that coagulation- and extracellular matrix–related pathways actively shape immune cell recruitment, stromal remodeling, and inflammatory signaling within the tumor microenvironment [27,28]. Similar immune-enriched microenvironmental features have been associated with aggressive CCA phenotypes and reduced therapeutic responsiveness [29].

Within a proof-of-concept framework, these findings are not intended to define a mechanistic role for SERPINF2 in immune modulation, but rather to illustrate how immune context can be integrated into early-stage biomarker prioritization to enhance biological coherence and interpretability.

### 6.4. Stemness and Tumor Plasticity as an Additional Contextual Layer

Beyond immune microenvironmental influences, CCA biology is also shaped by stemness-enriched cellular subpopulations that contribute to tumor initiation, therapeutic resistance, invasion, and recurrence. Increasing evidence indicates that CCA contains heterogeneous cancer stem cell (CSC)-associated subsets characterized by markers such as EpCAM, CD133 (PROM1) [30], CD90 [31], LGR5, and CD13, with variability across histologic and molecular subtypes. These stemness-associated phenotypes are frequently linked to developmental and plasticity-related signaling pathways, including Wnt/β-catenin, Notch [32], Hedgehog [33], and Hippo/YAP1 [34], which collectively support cellular adaptability and tumor aggressiveness.

Although the present study did not directly evaluate CSC phenotypes or stemness-associated functional properties, the cross-model conservation and multi-compartment detectability of SERPINF2 observed in our EV-guided prioritization framework may be compatible with broader tumor plasticity programs that persist across heterogeneous tumor states. CSC-enriched populations are often implicated in extracellular matrix remodeling, niche adaptation, and intercellular communication—processes that intersect with coagulation and fibrinolytic pathways in the tumor microenvironment. In this context, SERPINF2-associated molecular patterns may warrant further investigation to determine whether they align with stemness-related signaling contexts or CSC-enriched subsets in CCA.

Importantly, this perspective is not intended to define SERPINF2 as a CSC marker, but rather to situate its consistent deregulation within the broader biological landscape of CCA heterogeneity and cellular plasticity. Dedicated functional studies will be required to clarify whether SERPINF2 expression intersects with stemness-associated regulatory networks or influences CSC-related phenotypes.

### 6.5. Translational Signal Filtering Across Biofluids

A defining aspect of this proof-of-concept study is the exploratory extension from tumor-intrinsic and tissue-level analyses to clinically accessible biofluids. While tissue-level RNA-seq analyses demonstrated robust discriminatory performance for SERPINF2, biofluid EV analyses were intentionally designed as hypothesis-generating.

The contrasting detection patterns observed in serum- and urine-derived EVs are particularly informative. SERPINF2 was undetectable in serum EVs from both healthy donors and CCA patients, whereas it was detectable in urine-derived EVs from healthy individuals but absent in CCA urine EVs. Rather than being interpreted as a negative or inconclusive result, this differential detectability highlights a critical translational insight: distinct biofluids capture different EV populations and selectively filter disease-associated signals. Urine EVs, characterized by lower protein complexity and reduced interference from high-abundance plasma proteins, have increasingly been recognized as a promising non-invasive matrix for cancer biomarker discovery [35,36].

From a PoC perspective, this observation demonstrates how EV-guided discovery can inform biofluid selection early in biomarker development, prior to large-scale patient validation. Although limited by pooled samples, the directional consistency between EV proteomics, tissue-level expression, and urine EV detectability provides preliminary translational support for SERPINF2 and, more broadly, for the proposed framework.

## 7. Limitations

Several limitations of this study should be acknowledged. First, this work was designed as a proof-of-concept investigation rather than a definitive clinical validation study. As such, analyses of human biofluid-derived EVs were exploratory and performed using pooled serum and urine samples, which precluded assessment of inter-individual variability, statistical power, and biomarker performance metrics such as sensitivity and specificity. These analyses were intended to examine directional consistency and translational feasibility rather than to establish clinical utility.

Second, the study relied primarily on in silico integration of public transcriptomic, epigenetic, and regulatory datasets to infer mechanisms underlying SERPINF2 dysregulation. While convergence across multiple independent data layers strengthens biological plausibility, experimental validation of epigenetic regulation, miRNA targeting, and alternative promoter usage was beyond the scope of this work. Accordingly, these findings should be interpreted as hypothesis-generating rather than mechanistic confirmation.

Third, immune associations were derived from computational deconvolution of bulk transcriptomic data and should be interpreted as contextual rather than causal. Functional studies addressing the role of SERPINF2 in immune modulation, fibrinolysis–extracellular matrix remodeling, or tumor–immune interactions will be required to establish mechanistic links.

Fourth, although multiple biologically distinct CCA cell models were incorporated to address tumor heterogeneity, cell line-based systems cannot fully recapitulate the complexity of patient tumors or their microenvironments. Accordingly, future studies should prioritize patient-level validation in independent cohorts, longitudinal assessment of urine EV-associated SERPINF2, and evaluation of this marker in combination with established CCA biomarkers.

Finally, EVs were isolated using differential ultracentrifugation, which may permit partial co-isolation of lipoproteins and other high-abundance proteins, particularly in serum-derived samples. Although washing steps were implemented to reduce soluble protein carryover, marker-based validation of EV-positive and EV-negative proteins was not performed for biofluid-derived preparations. Therefore, these samples should be interpreted as EV-enriched fractions rather than highly purified vesicle populations. As biofluid EV analyses were conducted primarily as a translational detectability screening step within a proof-of-concept prioritization framework, future studies incorporating advanced purification strategies and expanded validation cohorts will be necessary to further substantiate these findings.

In addition, the observed AUC of 1.00 for SERPINF2 derived from TCGA-CHOL versus GTEx comparisons should be interpreted with caution. Cross-cohort integration of independently generated datasets may be influenced by residual batch effects despite standardized normalization procedures within public platforms. Therefore, independent cohort validation and harmonized batch-correction approaches will be required to confirm the robustness and generalizability of this observation.

Despite these limitations, the present study achieves its intended proof-of-concept objective by establishing an EV-guided, multi-compartment framework for biomarker prioritization. This framework is designed to reduce downstream translational attrition by aligning molecular robustness, compartment-specific detectability, and translational feasibility at the discovery stage.

## 8. Future Directions

Future studies should primarily focus on further evaluating and refining the proposed EV-guided, multi-compartment discovery framework rather than on the validation of a single biomarker candidate. Applying this framework to additional EV-associated proteins will allow systematic assessment of whether compartment-specific signal preservation, loss, or filtering represents a generalizable phenomenon rather than a SERPINF2-specific observation.

Prospective application of this approach across independent cell models, tumor types, and biofluid contexts will help define the reproducibility and boundary conditions of the framework. In particular, comparative analyses across multiple biofluids may clarify how biological compartmentalization influences EV cargo detectability and translational relevance, thereby informing rational biofluid selection at an early discovery stage.

Beyond CCA, this EV-guided, multi-compartment strategy may be extended to other heterogeneous malignancies where conventional biomarker discovery approaches frequently fail due to poor transferability from tumor tissue to clinically accessible samples. Integration with longitudinal or treatment-associated sampling could further explore how EV-associated molecular features behave across disease states, providing insight into whether this framework can support dynamic biomarker prioritization rather than static candidate identification.

Collectively, these future efforts will help establish whether EV-guided, compartment-aware discovery can serve as a broadly applicable strategy for reducing attrition in biomarker development, independent of individual molecular candidates.

## 9. Conclusions

This study presents an EV-guided, multi-layer, multi-compartment proof-of-concept framework that advances biomarker discovery beyond conventional single-context strategies. Traditional approaches often prioritize differential expression within isolated sample types, implicitly assuming uniform signal transfer from tumor tissue to clinically accessible biofluids. In contrast, our framework explicitly evaluates how molecular signals are preserved, filtered, or lost across biologically and translationally distinct compartments.

By integrating EV exportability, tolerance to tumor heterogeneity, multi-layer regulatory coherence, and compartment-aware signal assessment, this approach enables earlier discrimination between biomarkers that are merely tumor-associated and those more likely to retain clinically interpretable signals. Importantly, the contribution of this work lies not in validating a single candidate biomarker, but in establishing a scalable and concept-driven strategy that addresses a key source of translational attrition in biomarker research.

As such, this EV-guided framework provides a practical and generalizable blueprint for biomarker prioritization in heterogeneous malignancies, offering a rational alternative to conventional discovery pipelines that often struggle to bridge molecular relevance with clinical feasibility, without requiring large-scale upfront validation.

## Figures and Tables

**Table 1:** The IC_50_ [median (range)] and SI values of cisplatin for CL-6, HuCCT-1, HuH-28, and MMNK-1 cells.

Cell Lines	Cisplatin Concentration (µg/mL)	SI
	IC_50_	
CL-6	5 (4.50–5.37)	0.54
HuCCT-1	8 (7.46–9.1)	0.34
HuH-28	55 (51.01–57.9)	0.05
MMNK-1	2.7 (2.65–3.0)	-

## Data Availability

The raw proteomics data generated and analyzed during this study have been deposited in the jPOST (Japan Proteome Standard Repository) under the accession number JPST004154 “https://proteomecentral.proteomexchange.org/cgi/GetDataset?ID=PXD070190 (accessed on 21 February 2026) ” and PXD070190.
